# Ageism against older adults in the context of the COVID-19 pandemic: an integrative review

**DOI:** 10.11606/s1518-8787.2021055003082

**Published:** 2021-04-05

**Authors:** Marcela Fernandes Silva, Diego Salvador Muniz da Silva, Aldiane Gomes de Macedo Bacurau, Priscila Maria Stolses Bergamo Francisco, Daniela de Assumpção, Anita Liberalesso Neri, Flávia Silva Arbex Borim

**Affiliations:** I Universidade Estadual de Campinas Faculdade de Ciências Médicas Departamento de Gerontologia CampinasSP Brasil Universidade Estadual de Campinas. Faculdade de Ciências Médicas. Departamento de Gerontologia. Campinas, SP, Brasil; II Universidade Estadual de Campinas Faculdade de Ciências Médicas Departamento de Saúde Coletiva CampinasSP Brasil Universidade Estadual de Campinas. Faculdade de Ciências Médicas. Departamento de Saúde Coletiva. Campinas, SP, Brasil; III Universidade de Brasília Faculdade de Ciências da Saúde Departamento de Saúde Coletiva BrasíliaDF Brasil Universidade de Brasília. Faculdade de Ciências da Saúde. Departamento de Saúde Coletiva. Brasília, DF, Brasil

**Keywords:** Aged, Ageism, Health of the Elderly, Geriatrics, Coronavirus Infections, Social Discrimination, Prejudice, Stereotyping, Health Policy, Review

## Abstract

**OBJECTIVE:**

To report the main results of studies on prejudice, stereotyping, and age-based discrimination (ageism) in the context of the COVID-19 pandemic.

**METHODS:**

This is an integrative review of the literature on ageism in the context of the COVID-19 pandemic, conducted between May and June 2020, with data collected from the following databases: Medical Literature Analysis and Retrieval System Online (MEDLINE/PubMed), Web of Science (Thompson Reuters), Scopus (Elsevier Science), *Literatura Latino-Americana e do Caribe em Ciências da Saúde* (Lilacs) and Scientific Eletronic Library Online (SciELO).

**RESULTS:**

Twenty-one publications addressing ageism during the pandemics, its origins, consequences, and ethical and political implications were analyzed. All publications were theoretical with a critical/reflexive approach, being 90,5% opinion articles (n = 19) and 9,5% research (n = 2). The main findings indicate criticisms regarding resources allocation and intensive care based exclusively on age. The results also highlight the impacts of social isolation, the use of technologies and social media, and intergenerational relationships within the COVID-19 scenario.

**CONCLUSION:**

According to most publications, although ageism has always been present, it became more evident during the COVID-19 pandemic as a form of discrimination against older adults. “Ageist” discourses may exert a negative influence in older adults’ lives, causing severe social and psychological impacts.

## INTRODUCTION

In December 2019, a severe respiratory disease of unknown etiology was detected in the city of Wuhan, China. Later on, it was identified as an infectious disease caused by the novel coronavirus (Severe Acute Respiratory Syndrome Coronavirus-2 or Sars-Cov-2) and named coronavirus disease 2019 (COVID-19)^1^. Due to the rapid spread of the virus and the growing number of cases worldwide, the World Health Organization (WHO) declared COVID-19 a global pandemic on March 11, 2020^2^.

The novel coronavirus pandemic is one of the biggest public health issues over the last century, posing challenges such as the implementation of measures that ensure social health protection and minimize economic and social damage while respecting human rights^3^. Although everyone is susceptible to the disease, countries with older populations have felt the impacts of the pandemic in a larger scale, especially regarding morbidity and mortality. Studies show that older adults are at higher risk of developing severe forms of COVID-19, possibly leading to death^4^. Among other factors, this may be explained by immunosenescence – a process characterized by the gradual deterioration of the immune system and the consequent increased susceptibility to infections^6^. Besides age, the high prevalence of multimorbidity, frailty, and inflammatory changes make this age group more vulnerable and may complicate the disease course^7^.

As a measure to contain the pandemic expansion, the WHO suggested all countries to adopt social distancing measures to contain virus spread, prevent health systems collapse, and reduce the number of victims of COVID-19^8^. However, this strategy may generate negative impacts in various segments of society. Bezerra et al.^9^ conducted an opinion survey on perceived social isolation during the COVID-19 pandemic in Brazil (n = 16,440) and found social interaction to be the most affected aspect, followed by financial condition.

Global health authorities then began to recommend other strategies to reduce virus transmission, such as prohibiting agglomerations, restricting movement, and limiting contact with special populations (as in long-term institutions, such as prisons). Such measures incur social, economic, and health-related consequences^10^.

Various age groups may be vulnerable to the effects of COVID-19 control and preventive measures, including the social distancing and isolation stemming from them. Nowadays, older adults are increasingly likely to live alone and have fewer opportunities for social interaction^11^, besides going out less often for social, recreational, religious, and utilitarian activities due to mobility difficulties and inadequate environmental conditions. This population also uses less online communication systems to inform themselves, shop, contact other people, and have fun when compared to younger individuals. With that, older adults are particularly exposed to the risks arising from social isolation and loneliness enforced by social distancing measures^12,13^.

Social isolation is the absence of social contact or communication, participation in social activities, or confidants contact, increasing the risk of death by almost one third (OR = 1.29)^14^. Often associated with social isolation^13,15^, emotional loneliness is a personal experience of lack of significant social connections that evokes negative emotions such as disinterest, boredom, fatigue, and apathy, besides potentiating pain, sleep disorders, decreased appetite, and physical inactivity. Together, the consequences of social isolation and emotional loneliness in older adults increase their vulnerability to depression and risk of death^15,16^.

The COVID-19 pandemic and the consequent risk of overloading health systems in some countries and regions sparked discussions on health resources allocation primarily to young and adult patients. This possibility provoked and fueled the controversy surrounding fundamental ethical issues, including the right to life and professional’s decision on who lives and who dies^17^. Concurrently, derogatory memes, negative stereotypes, and biased discourses against older adults featured on the Internet, media, and social networks, evincing age-based discrimination in society^18^.

That is, the pandemic brought to light the issue of prejudice toward older populations, which is not recent in history. The term “ageism” was first used in 1969 by the American psychiatrist and gerontologist Robert Butler to designate the prejudice by one age group toward other age groups, or as any form of stereotyping and discriminating people based on their chronological age^19^. Robert Butler also emphasizes ageism essential vocations – being oriented toward older adults and including systematic processes of stereotyping and discriminating people due to their age. The author classified ageism as a form of intolerance comparable to sexism and racism. Six years later, he refined the concept by stating that ageism includes biased attitudes toward older people, old age, and the aging process; discriminative social practices against older adults; and institutional practices and policies that perpetuate stereotypes against these age groups^20^.

Ageism may be perpetuated against young people and adults^21,22^, but most theoretical studies and research on the theme focus on older adults. This is particularly true regarding the treatment provided to older populations during the COVID-19 pandemic, considering this group greater biological vulnerability and lower political power when compared to younger groups. The term ageism was translated to Brazilian Portuguese as *discriminação por idade, etarismo,* or *ageismo,* and recorded in the list of Descriptors of Health Sciences (DeCS) of the Latin American & Caribbean Health Sciences Literature (Lilacs) and the Virtual Health Library (VHL).

Considering aging as a complex, dynamic, and heterogeneous process, discrimination against older adults and age-based stigmatization have been more evident in view of the COVID-19 pandemic, requiring major ethical and political discussions. This review sought to describe the main results of studies on prejudice, stereotyping, and age-based discrimination (ageism) in the context of the COVID-19 pandemic.

## METHODS

The integrative literature review is a methodological approach used to provide knowledge on a given theme in a systematic, orderly, and comprehensive manner. It is organized into six phases: identifying the theme, hypothesis, or research question; identifying in the literature pre-established search criteria; defining information to be extracted from selected studies/categorizing studies; evaluating studies critically; interpreting results; and presenting the review/synthesis of knowledge^23^.

This study conducted a literature search on national and international journals addressing age discrimination (ageism) against older adults in the context of the COVID-19 pandemic. Between May 1 and June 15, 2020, two independent researchers conducted the electronic search in to the following databases: Medical Literature Analysis and Retrieval System Online (MEDLINE/PubMed), Web of Science (Thompson Reuters), Scopus (Elsevier Science), Lilacs, and the Scientific Eletronic Library Online (SciELO). To solve divergences, a third researcher was consulted for an opinion on whether to include or not the selected publications.

Descriptors were used according to the Medical Subject Heading (MeSH) and its Portuguese equivalents, provided by the Descriptors in Health Sciences (DeCS). The search strategy, elaborated for each database, comprised terms combined using the Boolean operators “AND” and “OR.” The following terms were used as descriptors for database search: covid-19 OR 2019 novel coronavirus disease OR covid19 OR covid-19 pandemic OR SARS-CoV-2 infection OR covid-19 virus disease OR 2019 novel coronavirus infection OR 2019-nCoV infection OR coronavirus disease 2019 OR coronavirus disease-19 OR 2019-nCoV disease OR covid-19 virus infection AND Ageism OR Discrimination OR Age Discriminations OR Discrimination, Age OR Discriminations, Act.

All publications approaching ageism in older adults (people aged ≥ 60 years) and its impacts on the COVID-19 pandemic were eligible for inclusion. We applied no restrictions regarding year, methodological design, or language. Articles discussing ageism in age groups below 60 years or unrelated to the COVID-19 pandemic were excluded. Publications duplicated in different databases were considered only once.

## RESULTS

The initial search identified 43 publications based on the title. After title and abstract screening, 21 were excluded for being duplicate or unrelated to the theme of interest. Twenty-two studies were selected for full-text reading, from which one was excluded after consultation by the third researcher for not addressing ageism in the pandemic as main theme. Thus, this integrative review comprised 21 articles. Of these, 18 were identified in the MEDLINE/Pubmed, seven in the Web of Science, four in the VHL/Lilacs, and three in Scopus. Some articles were published in two or more databases. Figure 1 shows the flowchart outlining the process for selecting the publications.

**Figure:**
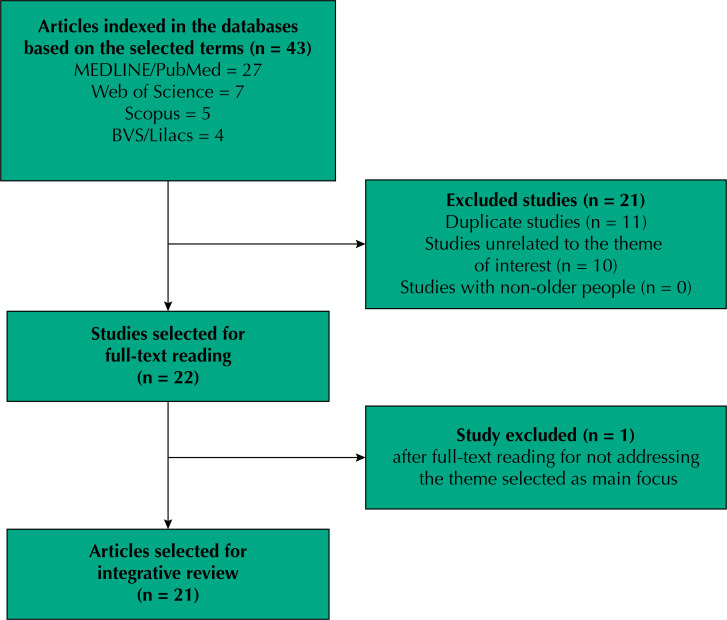
Flowchart of the selection process for studies included in the systematic review on ageism in the context of the COVID-19 pandemic.

Chart 1 synthesizes the publications contemplated in this review according to database, journal, author(s), publication year, title, type of study, considerations/objectives, and the results of interest. Nineteen articles (90,5%) were published in English, one in Spanish and one Portuguese. All publications were theoretical with a critical/reflexive approach, being 90% opinion articles (n = 19) and 9,5% research (n = 2).

**Chart:** Integrative review of publications on ageism against older adults in the context of the pandemic caused by SARS-CoV-2 (COVID-19).

Database	Journal	Author(s), publication year	Title	Type	Objectives	Results^a^ / Contents^b^
PubMed, Web of Science	Journal of Clinical Nursing	Brooke et al., 2020^18^	Older people and COVID-19: isolation, risk and ageism^b^	Editorial	It addresses social isolation, risks, and ageism during COVID-19.	Biased, negative, and implicitly devaluing discourses contribute for older adults to feel useless, burdensome, and valueless, diminishing their social opportunities and making them vulnerable to negative social and health outcomes, especially isolation and loneliness.
PubMed, BVS, Scopus, Web of Science	Journal of the American Medical Directors Association	Cesari et al., 2020^24^	COVID-19 in Italy: Ageism and decision making in a pandemic^b^	Editorial	It criticizes ethical and clinical guidelines regarding the allocation of scarce intensive care resources based on the age criterion during the COVID-19 pandemic in Italy.	Conducts driven only by the number of years lived configure ageism. Critical and rapid decision-making in older people’s health require parameters more robust than age, including frailty, comorbidities, and functional status.
PubMed, Web of Science	Journal of the American Geriatrics Society	Jimenez-Sotomayor et al., 2020^25^	Coronavirus, ageism, and twitter: an evaluation of tweets about older adults and COVID -19^a^	Qualitative research	It analyzes tweets related to older adults and COVID-19 to identify of age-based discrimination content.	Almost a quarter of the tweets analyzed (21.1%; n=74) addressed age-based discrimination content or was potentially offensive to older people, designated as “boomers.” The comments diminished the value of older adults’ lives or minimized COVID-19 hazards for affecting this age group.
PubMed	Geriatrics	Petretto et al., 2020^26^	Ageing and COVID-19: what is the role for elderly people?^b^	Editorial	It addresses the role of older adults in the context of COVID-19 and highlights the risks of ageism from the Italian experience.	Older adults’ greater vulnerability to coronavirus increases the risk of ageism, and actively performing roles during the pandemic reduces such risk. As a result of ageism, older adults may face major barriers in accessing healthcare and support. Protecting older adults from infection is important, but so is respecting and supporting them in this complex situation.
PubMed, Web of Science	Journal of Aging & Social Policy	Morrow-Howell et al., 2020^27^	Recovering from the COVID-19 Pandemic: a focus on older adults^b^	Article – Perspective	It discusses the challenges (including ageism) and opportunities arising from the COVID-19 pandemic based on the experience of older adults.	The COVID-19 pandemic revealed that ageism and old age-based stereotypes contributed to abandoning older adults and neglecting their needs. Memes and hashtags that disseminate the idea of eliminating or getting older adults out of the way depreciate them and validate the virus by stimulating a reduced public expenditure with this age group – deemed as unproductive, dependent, and expensive.
PubMed	Age and Ageing	Fraser et al., 2020^28^	Ageism and COVID-19: what does our society’s response say about us?^b^	Commentary	It stresses the ageism arising from the COVID-19 pandemic and discusses how older people are misrepresented and devalued in the current public discourse around the pandemic.	The current public discourse around COVID-19 misrepresents and devalues older people. At first, the pandemic was not taken seriously and was portrayed by the public discourse as only dangerous for older adults. This narrative might explain the social resistance in following public health recommendations. Ageism has reached a new level with memes and hashtags that talk about killing or eliminating older adults, represented as vulnerable and helpless beings whose death by the virus was predicted and inevitable.
PubMed, Scopus	Asian Journal of Psychiatry	Banerjee, 2020^29^	‘Age and ageism in COVID-19’: elderly mental health-care vulnerabilities and needs^b^	Note to the Editor	It addresses ageism and age vulnerability in the COVID-19 pandemic and the need for promoting mental healthcare and the well-being of older adults.	The impact of the pandemic may be greater in older adults, given they are aware of their vulnerability and may self-neglect. Ageism may lead to elder marginalization and abuse, fostering functional dependence and decreasing well-being.
PubMed	Journal of Gerontological Social Work	Berg-Weger et al., 2020^30^	COVID-19 Pandemic: Workforce implications for gerontological social work^b^	Article	It addresses four issues that became more pronounced with the pandemics: ageism, technology, social isolation and loneliness, and interprofessional practice, considering workforce implications for gerontological social work with COVID-19.	Different ageist conducts were demonstrated throughout the pandemic, including the lack of protocols for older adults and gerontological content in the curricula of health professions providing care to older adults, inequalities in resources allocation, derogatory references to older adults, and relief for this age group presenting more risk of mortality.
PubMed, Web of Science	Journal of Gerontological Social Work	Swinford et al., 2020^31^	Applying gerontological social work perspectives to the coronavirus pandemic^b^	Commentary	It analyzes three gerontological perspectives of social assistance during the COVID-19 pandemic.	The older population heterogeneity challenges ageism and the age-based stereotypes that emerged with COVID-19. Ageism notions are based on harmful stereotypes that reduce a group extremely heterogeneous into a single cohort based on their age. Rejecting ageist discourses and reinforcing arguments to support older adults and workers, volunteers and caregivers, are important measures, as well as actively promoting efforts to foster solidarity between generations.
PubMed	International Psychogeriatrics	Ayalon, 2020^17^	There is nothing new under the sun: ageism and intergenerational tension in the age of the COVID-19 outbreak^b^	Commentary	It addresses the relation between ageism and intergenerational tension in the COVID-19 pandemic.	Older adults were the main affected and less compliant with social practices implemented during the pandemic. All older adults were placed in a same homogeneous, vulnerable group, disregarding the heterogeneity of aging and reinforcing ageism, which sparks disagreements between different generations.
PubMed	The Journals of Gerontology	Ayalon et al., 2020^32^	Aging in times of the COVID-19 pandemic: avoiding ageism and fostering intergenerational solidarity^b^	Editorial	It expatiates on how ageist discourse can affect all generations.	The pandemic provoked a parallel outbreak of ageism. The media depicted older adults as a fragile group, made of powerless people, unable to contribute to society. Incorporating these stereotypes may be detrimental for both older adults and young people, during their own aging. Physical distancing must not imply social distancing, and relationships between generations must be strengthened. The concept of risk group should take into account other factors besides age, such as chronic diseases and frailty.
PubMed, BVS, Scopus	British Medical Journal	Archard et al., 2020^33^	Is it wrong to prioritize younger patients with covid-19?^b^	Note to the Editor (Opinion)	It explains the reasons why age cannot be a determining factor in deciding which life should be prioritized.	Everyone has the right to live for a certain duration, so that deciding who gets to live based only on age is not right. Moreover, old lives worth just as much as young ones. The key point is employing ethics to avoid having discriminatory and ageistic attitudes – according to which older adults worth less or are less important than young people.
PubMed	Journal of Aging & Social Policy	Ehni et al., 2020^34^	Six propositions against ageism in the COVID-19 pandemic. ^b^	Article (other category)	Based on gerontological knowledge and the ethics of aging, it offers six proposals against the ageism pervading the current reactions to the COVID-19 pandemic.	The pandemics evinced comments on older adults developing the most severe form of COVID-19 and having a greater risk of dying. During the pandemics, many attitudes are based on negative stereotypes of older adults’ health and functioning, devaluing their lives and exacerbating ageism. Considering that, the authors offer six proposals to change such behavior, which will be presented later.
PubMed, BVS	*Revista Española de Geriatria y Gerontologia*	Tarazona-Santabalbina et al., 2020^35^	COVID-19, adult mayor y edadismo: errores que nunca han de volver a ocurrir [COVID-19, olderadults and ageism: mistakes that should never happen again]^b^	Editorial	It discusses ageist attitudes during the pandemic and what must be done to tackle them.	Decisions made during this health emergency do not justify the devaluation of older people. Geriatrics and gerontology professionals must join efforts to disclose more information and terminate ageism. Older adults must also have access to tests and specialized health teams to avoid such mistakes in the future.
PubMed	British Journal of Anaesthesia	Savulescu et al., 2020^36^	Equality or utility? Ethics and law of rationing ventilators^b^	Editorial	It explains the ethical terms egalitarianism and utilitarianism and how they apply to decisions regarding the allocation of intensive care and mechanical ventilation and the lives to be saved in the pandemic scenario.	Decisions regarding mechanical ventilation should be made by the health team together with the patient and grounded on aspects other than simply age or disability – as this could be jaundiced. Recognizing that people should not be arbitrarily discriminated, the authors suggest using “preventive utilitarianism” to ensure equality, so that the largest number of people would benefit from the greatest good in an equal manner.
PubMed	Journal of Aging & Social Policy	Previtali et al., 2020^37^	Not only virus spread: The diffusion of ageism during the outbreak of COVID-19. ^b^	Article	It addresses ageism diffusion during the COVID-19 pandemic.	Ageism has harmful effects on society and its incidence has increased during the pandemic. Although COVID-19 affects all age groups, older populations have been highlighted by the media. Several social media comments reinforce ageism and, contrary to what has been disclosed, older adults are highly active in society and extremely affected by social isolating measures. Ageist practices during the pandemics reinforce stereotypes and violate human rights, demanding a collective effort to end this.
PubMed	European Cardiology Review	Martínez-Sellés et al., 2020^38^	Ethical issues in decision-making regarding the elderly affected by coronavirus disease 2019: an expert opinion^b^	Opinion article	It expatiates on decisions regarding the older population during the COVID-19 pandemic.	Older adults should be prioritized in preventive measures against coronavirus given they are at higher risk of contamination. Yet, decisions should not be grounded solely on age, but rather consider those more likely to survive. Social isolation incur physical and psychological risks to older adults, not only within their household, but also in hospitals, institutions, and even at wakes.
PubMed	Journal of Aging & Social Policy	Reynolds, 2020^39^	The COVID-19 pandemic exposes limited understanding of ageism^b^	Article	It addresses the lack of knowledge on ageism and its impacts on the ageing process.	With the COVID-19, the lack of knowledge regarding ageism was very evident. An important example of ageism dimensions and constructions in the context of COVID-19 was the case of the Lieutenant Governor of Texas, who said he would give up his own life to save his grandchildren’s generation. Health professionals and caregivers also state unintentional ageist comments. We must implement this biopsychosocial concept within different spheres.
BVS	*Cogitare Enfermagem*	Hammerschmidt, et al., 2020^40^	*Saúde do idoso em tempos de pandemia COVID-19*^b^	Free communication	It addresses, in a reflexive and critical manner, aspects related to older adults’ health in pandemic times.	The pandemic bloomed ageism. Measures aiming to protect older adults during the pandemics reinforced age-based stereotypes. This situation may have negative impacts on family relationships and promote intergenerational conflicts. Despite highlighting the importance of healthcare for older adults, the COVID-19 pandemic also reinforced ageist behaviors, mockery, and judgments. Physical distancing actions should maintain the autonomy and independence of older people.
Web of Science	Journal of Loss and Trauma	Rahman, et al., 2020^41^	Defining a ‘risk group’ and ageism in the era of COVID-19^b^	Article	It deals with the COVID-19 cumulative incidence, addressing the classification of the group at greater risk, which is not only questionable, but also problematic.	Labeling older adults as a risk group relying entirely on age is a form of ageism that may accelerate social isolation and increase levels of psychosocial pain. Besides age, social, cultural, and contextual factors must be taken into account for formulating these groups. Social media platforms should be used positively to spread facts and avoid panic. The heterogeneity inherent to the aging process must be recognized, and positive attitudes toward older adults must start within our homes and enter digital media.
Web of Science	International Journal of Mental Health and Addiction	Flett, et al., 2020^42^	Aging and feeling valued versus expendable during the COVID-19 pandemic and beyond: a review and commentary of why mattering is fundamental to the health and well-being of older adults^a^	Review and comment	It assesses the protective role of feelings considered important for older people in typical and atypical times, such as the COVID-19 pandemic.	The authors present the concept of “mattering*”* and its particularities in the older population. All people want to feel important and valued, and this fact is even more pronounced among older adults during the pandemic. Many older adults may feel more vulnerable and helpless as a result of government recommendations and the comments made. Social isolation likewise causes further hazards to the health of this population. Considering that, biased comments on aging should be avoided and measures should be developed to preserve the physical and mental health of older people.

^a^ Research articles.

^b^ Opinion articles.

Opinion pieces outlined the importance of researchers to position themselves regarding a global bioethical, cultural, social, and ethical issue. By doing that, the authors take a stand not only as health professionals, but also as human beings before their equals, experiencing an issue that will scar many people’s fate. The novel coronavirus bursts a new paradigm for searching for knowledge, common good, and solidarity.

Most studies^18,24,27^indicate that ageism was more evident during the COVID-19 pandemic, causing several negative impacts for older adults. Brooke e Jackson^18^ stress that prolonged isolation may lead to loneliness, decreased mobility, increased frailty, and depression among older persons. Besides that, “ageist” discourses may increase elder abandonment^18^. According to Banerjee^29^, elder neglect, loneliness, depression, anxiety, isolation, and abuse are evils associated with social distancing during the pandemic. Such scenario may be even more problematic among institutionalized adults, when distancing and hygiene measures may be inadequate. Morrow-Howell et al.^27^ stress that older persons may have long-term emotional effects due to increased isolation and anxiety.

By analyzing a series of tweets, Jimenez-Sotomayor et al.^25^ found 21.1% to contain age-biased comments or underestimate COVID-19 severity for believing it would only affect older groups. Other studies^17,18,28^ also verified ageism in social networks by the hashtag #boomerremover, often followed by derogatory images and jokes related to older persons. Some publications^24,33,36,40^. critically addressed resources allocation, intensive care, mechanical ventilation, and/or decision-making based exclusively on the age criterion.

The studies also addressed the issue of ageism and intergenerational relationships^17,32^. “Ageist” discourses were increasingly present in the media during the pandemics, provoking conflicts between people of different generations. Social stratification by age makes it even more difficult for older adults to cope with a devastating natural disaster such as the pandemic^17^. Health professionals likewise reinforced the emphasis on age as a determining factor of COVID-19 severity, highlighting the knowledge gap on ageism by them and the general population. Although rarely intentional, ageism may ensue negative consequences for older persons’ lives. Considering that, understanding ageism as a biopsychosocial concept and disseminating its definition through different spaces is crucial^41^.

## DISCUSSION

Our main findings show that ageism has become present in different aspects of the lives of older adults during the COVID-19 pandemic. Although everyone is vulnerable to the novel coronavirus, older adults are at the core of the media and in most discussions about the theme.

A study^42^ found that ageism experiences were more common among older and young adults than in middle-aged people. While young adults reported experiencing ageism more often within their workplace, older and middle-aged individuals suffered it when looking for goods and services. For older persons, family members were not the main practitioners of ageism – although comments of this type made by a family member are interpreted as less aggressive. Regarding experiences, young people witnessed lack of respect whereas middle-aged and older adults were victims of assumptions regarding their social and physical capacity^42^.

Ageism may occur at the structural level, whereby social institutions reinforce systemic discrimination against older persons, or at the individual level, whereby individuals have negative opinions regarding ageing. A systematic review with 422 studies (including over 7 million participants) found ageism to lead to poorer health outcomes in older adults in 95.5% of the studies. Moreover, less developed countries presented a higher prevalence of the negative effects of ageism than more developed countries (p = < 0.001), so that it was associated with decreased health in all domains analyzed^43^.

Given that many older adults have to remain within their households, depending on other people’s help and service provision to obtain, for example, basic items, ageism may occur more frequently during the pandemic. In that way, studies may misfocus older adults’ physical characteristics, categorizing them as a heterogeneous group of frail and dependent people^36^.

A study conducted with older adults from Australia^44^found those presenting with some type of disability to be more likely to report discrimination than those without disability or with chronic diseases. Victims of prejudice reached lower scores of self-efficacy and life satisfaction, evincing the negative effects of discrimination on older adults’ lives^44^.

Previtali et al.^37^ emphasize that, despite the association between chronic diseases and age, being chronologically older does not presuppose being vulnerable or less valuable or having a precarious health. The notion that chronological age objectively defines groups, neglecting their internal differences, is an ageist assumption that supports age-based prejudices, stereotypes, and discrimination^37^.

Some studies report the presence of ageism in health services during the COVID-19 pandemic based mainly on resources allocation, such as prioritizing the allocation of mechanical ventilators for young people in detriment to older people. Due to the large and growing number of COVID-19 patients in Italy, the country began to implicitly adopt the age criterion for deciding the allocation of scarce resources^24^. According to Ouchida^45^, how health professionals cope with the aging process and the older adult may determine their medical assistance and treatment. Resources allocation based solely on age characterizes ageism, given that other parameters should be considered in critical situations, such as clinical conditions, frailty, functional status, and comorbidities^24^. Everyone has the right to life, and quick decisions should be made by the health team together with the patient and family^33,36^.

Ageism may be either implicit or explicit and might not be recognized as such. As a result of the growth in the world’s older population, an adequate communication between health professionals and individuals, a better understanding of aging heterogeneity, and the resignation of age-related stereotypes are increasingly important^46^. For Cesari et al.^24^, physicians familiarized with geriatrics and gerontology principles must help formulating more contemporary recommendations, identifying valid and efficient ways to assess morbidities and functional status in different contexts and specialties.

The publications analyzed indicate that social isolation has a negative impact on older adults’ lives. Plagg^47^ evaluated the benefits and harms caused by long-term social isolation and found that, despite primarily aiming to avoid or reduce virus spread, this situation increased the risk of neurological and cardiovascular diseases, depression, cognitive decline, and mortality among older adults^45^. Thus, authorities must implement measures to reduce possible harms in the case of long-term social isolation. Distancing does not implicate the termination of social relationships and support networks, and health professionals, family, and society as a whole should work together to nurture the feeling of belonging among older adults.

With physical distancing and home isolation recommendations, social media emerged as the main alternative for individuals to keep some human interaction, even if indirect. Media coverage of the COVID-19 pandemic played a key role in quick disseminating scientific research and information from health authorities. Conversely, fake news, derogatory memes, and offensive opinions spread (such as the hashtag #boomerremover) evinced the ageism prevailing in society, which misconceives coronavirus as a disease “of the old” and potentiates the discriminatory content against older persons^25,27^. Creating reliable, high-quality content that confronts ageism is necessary to reduce the harmful effects of negative age-based stereotypes on older adults’ health and well-being^25,48^.

Intergenerational tension (the conflict between people of different generations) is manifested on social networks in the form of anger and hatred as a result of some older adults’ resistance in wearing masks or adhering to social isolation measures. Such social scenario also coined the notion that older individuals “have already lived their lives” and now it is time for them to resign, ignoring their autonomy and independence, and disregarding their social needs^17^. By relying solely on age as a risk and lethality marker for COVID-19, these facts reveal the sharp difference and animosity between generations^17^.

Stereotyping older adults as fragile and dependent may incur issues in all generations, as younger ones will internalize and project this image into their own aging process. Besides age, other factors make individuals more vulnerable to COVID-19, such as the presence of chronic diseases and comorbidities^32^. Solidarity between generations is important to maximize support, interaction, and the social support network of older individuals during the pandemic^28^. A systematic review and meta-analysis of 63 studies (6,124 participants) found that interventions focused on education, intergenerational contact, and the combination of education and intergenerational contact were associated with ageism reduction^49^.

Social behavior in old age is yet another aggravating factor, often characterized by reduced social networks and decreased participation in social activities^50^. Likewise, older adults’ limited access or capacity to use digital technologies may impede or hinder them from obtaining required goods, services, and social support during the pandemic, thus leaving them more vulnerable to isolation, depression, and loneliness^32^. For Previtali et al.^37^, access to technology and digital literacy were key elements for dealing with the challenges posed by quarantine.

Although protecting older adults from COVID-19 is crucial, respecting and supporting them in this complex situation is also important^26^. Considering the emergence of numerous ageist dilemmas and moral conflicts regarding the value of older people’s lives in the midst of the pandemic, Ehni et al.^34^ elaborated six aspects to combat ageism in pandemic reactions based on gerontological knowledge and the ethics of aging: 1) older adults are highly heterogeneous – their health and functioning are better than negative stereotypes suggest; 2) age limits for intensive care and other forms of medical care are inappropriate and unethical; 3) the deficient perspective regarding old age is dangerous for older adults and societies in general – intergenerational solidarity must be strengthened; 4) individuals must resist the assumption of a paternalistic attitude towards older groups; 5) the COVID-19 crisis requires older adults to use modern information and communication technologies; 6) for political orientation and for understanding the consequences of the COVID-19 crisis, not only the best of virology is necessary, but also that of gerontology.

Among the limitations of this study, we must consider that ageism in the context of the COVID-19 pandemic is still little discussed, which may explain the reduced number of publications addressing the theme and the higher prevalence of opinion pieces (editorials and comments). However, the critical-reflexive nature sustained by important theoretical assumptions enabled us to analyze several critical sociocultural aspects, comprehensive in public health.

Our results show an overview of the context and phenomenon addressed in the primary studies, bringing to light the issue of ageism, so important but little discussed in regard to the pandemic in Brazil and worldwide.

“Ageist” discourses may have negative social and psychological impacts on older adults’ lives, so that authorities should redouble efforts to reduce ageism and the dissemination of information on this harmful practice. Further public policies and scientific studies addressing the theme should be developed to promote a more just and egalitarian society, with intergenerational solidarity and respect for the rights and lives of the older people.
