# Supplementary material for: Ageism against older adults in the context of the COVID-19 pandemic: an integrative review
Source: Rev Saude Publica. 2021 Apr 5;55:4. doi: 10.11606/s1518-8787.2021055003082 (PMC8023321; doi:10.11606/s1518-8787.2021055003082)
Supplement: Translation

# Translation: Ageismo contra idosos no contexto da pandemia da covid-19: uma revisão integrativa

**Authors:** Marcela Fernandes Silva, Diego Salvador Muniz da Silva, Aldiane Gomes de Macedo Bacurau, Priscila Maria Stolses Bergamo Francisco, Daniela de Assumpção, Anita Liberalesso Neri, Flávia Silva Arbex Borim

**Affiliations:** I Brasil Universidade Estadual de Campinas. Faculdade de Ciências Médicas. Departamento de Gerontologia. Campinas, SP, Brasil; II Brasil Universidade Estadual de Campinas. Faculdade de Ciências Médicas. Departamento de Saúde Coletiva. Campinas, SP, Brasil; III Brasil Universidade de Brasília. Faculdade de Ciências da Saúde. Departamento de Saúde Coletiva. Brasília, DF, Brasil

**Keywords:** Idoso, Ageismo, Saúde do idoso, Geriatria, Infecções por Coronavírus, Discriminação Social, Preconceito, Estereotipagem, Política de saúde, Revisão

## Abstract

**OBJETIVO:**

Descrever os principais resultados de estudos sobre preconceito, estereotipia e discriminação relacionados à idade (ageismo) no contexto da pandemia da covid-19.

**MÉTODOS:**

Trata-se de uma revisão integrativa da literatura sobre o ageismo no contexto da pandemia da covid-19, realizada entre maio e junho de 2020, a partir das seguintes bases de dados: *Medical Literature Analysis and Retrieval System Online* (*MEDLINE/PubMed*), *Web of Science* (*Thompson Reuters*), *Scopus* (*Elsevier Science*), Literatura Latino-Americana e do Caribe em Ciências da Saúde (LILACS) e *Scientific Eletronic Library Online* (*SciELO*).

**RESULTADOS:**

Foram analisadas 21 publicações que discorreram sobre o ageismo durante a pandemia, suas origens, consequências e implicações ético-políticas. As publicações identificadas são de natureza teórica com abordagem crítico-reflexiva, sendo 90,5% artigos opinativos (n = 19) e 9,5% de pesquisa (n = 2). Os principais resultados encontrados apontam críticas em relação à destinação de recursos e cuidados intensivos baseados exclusivamente no critério etário. São também apontados os impactos do isolamento social, o uso das tecnologias e mídias sociais e as relações intergeracionais no cenário da covid-19.

**CONCLUSÃO:**

A maioria das publicações indicam que o ageismo sempre esteve presente, mas tornou-se mais evidente durante a pandemia da covid-19 como forma de discriminação contra idosos. Ressalta-se que discursos “ageistas” podem influenciar negativamente na vida dos idosos e causar impactos sociais e psicológicos prejudiciais.

## INTRODUÇÃO

Em dezembro de 2019, uma doença respiratória grave, de etiologia desconhecida, foi identificada na cidade de Wuhan, China. Posteriormente foi reconhecida como uma doença infecciosa causada pelo novo coronavírus (*Severe Acute Respiratory Syndrome Coronavirus-2* ou Sars-Cov-2) e denominada *Coronavirus disease 2019* (covid-19)^1^. Devido à rápida disseminação do vírus e ao aumento do número de casos da doença em diferentes partes do mundo, no dia 11 de março de 2020, a Organização Mundial de Saúde (OMS) declarou a vigência da pandemia da covid-19^2^.

A pandemia causada pelo novo coronavírus constitui um dos maiores problemas de saúde pública dos últimos 100 anos. Os desafios impostos aos países incluem a definição de medidas que garantam a proteção da saúde e minimizem os danos econômicos e sociais, respeitando os direitos humanos^3^. Toda população mundial é susceptível à doença, mas países com populações mais idosas têm sofrido mais os impactos da pandemia, especialmente em relação à morbimortalidade. Estudos demostram que idosos possuem maior risco de desenvolver formas graves da covid-19, podendo levá-los ao óbito^4^. Tal fato relaciona-se, entre outros fatores, à imunossenescência, processo caracterizado pelo declínio progressivo da função imunológica e consequente aumento da suscetibilidade às infecções^6^. Além da idade, outros fatores como a alta prevalência de multimorbidade, fragilidade e alterações inflamatórias tornam esse grupo etário mais vulnerável e podem complicar o curso da doença^7^.

Dentre as medidas para conter a expansão da pandemia, a OMS sugeriu que todos os países adotassem o distanciamento social a fim de conter a propagação do vírus, evitar o colapso dos sistemas de saúde e reduzir o número de vítimas da covid-19^8^. Contudo, essa estratégia pode gerar impactos negativos em diversos segmentos da sociedade. Bezerra et al.^9^ realizaram uma pesquisa de opinião no Brasil sobre a percepção do isolamento social durante a pandemia de covid-19 (n = 16.440), identificando o convívio social como aspecto mais afetado entre as pessoas, seguido pelo financeiro.

As autoridades sanitárias nos diferentes países passaram, então, a recomendar a adoção de outras estratégias para conter a transmissão do vírus, tais como: a proibição de aglomerações, restrição à circulação e limitação do contato com populações especiais (por exemplo, em instituições de longa permanência, como é o caso de prisões). Tais medidas geraram impactos negativos sociais, econômicos e relacionados à saúde^10^.

Vários grupos etários podem ser vulneráveis aos efeitos das medidas de controle e prevenção da covid-19, incluindo o distanciamento e o isolamento social dele resultante. Na atualidade, os idosos estão cada vez mais expostos à possibilidade de viver sozinhos e ter menos oportunidades de interação social^11^, além disso, saem menos de casa para atividades sociais, recreativas, religiosas e utilitaristas, em função de dificuldades de mobilidade e por condições ambientais inadequadas. Outro fator relevante é que essa população recorre menos do que os não idosos aos sistemas de comunicações *on-line* para se informar, fazer compras, contatos e se divertir. Com isso, tornam-se particularmente expostos aos riscos decorrentes do isolamento social e da solidão durante o período de distanciamento imposto pela pandemia^12,13^.

O isolamento social é definido como a falta generalizada de contato ou comunicação social, de participação em atividades sociais ou de contato com um confidente, estando associado ao aumento de quase um terço na chance de mortalidade (OR = 1,29)^14^. Comumente associada ao isolamento social^13,15^, a solidão emocional é uma experiência pessoal de falta de contatos sociais significativos, que dá origem a sentimentos negativos como o desinteresse, tédio, fadiga e apatia, além de provocar a potencialização de dores, problemas de sono, perda de apetite e inatividade física. Em conjunto, as consequências do isolamento social e da solidão emocional aumentam a vulnerabilidade dos idosos à depressão e os expõem a um maior risco de morte^15,16^.

Com a pandemia da covid-19 e o risco de sobrecarga dos sistemas de saúde em alguns países e regiões, surgiram discussões acerca da destinação de recursos de saúde prioritariamente para pacientes jovens e adultos. Tal possibilidade suscitou e alimentou a polêmica em torno de questões éticas fundamentais, entre elas o direito à vida e o direito do profissional de decidir quem deve viver e quem deve morrer^17^. Paralelamente, *memes* depreciativos, estereótipos negativos e discursos preconceituosos contra os idosos emergiram na internet, nas mídias e nas redes sociais, evidenciando a discriminação por idade na sociedade^18^.

A pandemia evidenciou a questão dos preconceitos com relação aos idosos, que não é recente na história. O termo “ageismo” é um neologismo originário da língua inglesa (*ageism*), criado e usado pela primeira vez em 1969 pelo psiquiatra e gerontólogo norte-americano Robert Butler, para designar o preconceito de um grupo de idade contra outros grupos de idade, ou como toda forma de estereotipia e de discriminação contra pessoas com base na idade cronológica^19^. No mesmo texto, Robert Butler enfatiza as vocações essenciais do ageismo, de ser orientado a idosos e de incluir processos sistemáticos de estereotipia e discriminação contra pessoas em razão de sua idade. O autor classificou o ageismo como uma forma de intolerância comparável ao sexismo e ao racismo e, seis anos mais tarde, refinou o conceito, dizendo que o ageismo inclui atitudes preconceituosas em relação às pessoas idosas, à velhice e ao processo do envelhecimento; práticas sociais discriminativas contra os idosos e práticas/políticas institucionais que perpetuam os estereótipos contra os mais velhos^20^.

Embora se admita que o ageismo possa ser dirigido a jovens e adultos^21,22^, a grande maioria dos estudos teóricos e das pesquisas sobre ageismo é centrada nos idosos. Essa ocorrência é particularmente verdadeira com relação ao tratamento dispensado aos idosos durante a pandemia da covid-19, o que ocorre em função da combinação entre maior vulnerabilidade biológica e menor poder político desse grupo quando comparado os mais jovens. O termo *ageism* foi traduzido como discriminação por idade, etarismo ou ageismo e registrado na lista de Descritores das Ciências da Saúde (DeCS) da Literatura Latino-Americana e do Caribe em Ciências da Saúde (Lilacs) e da Biblioteca Virtual em Saúde (BVS).

Diante do contexto da pandemia da covid-19 e considerando o envelhecimento um processo complexo, dinâmico e heterogêneo, a discriminação contra idosos e a estigmatização com base na idade têm sido mais evidentes, o que demanda discussões éticas e políticas importantes. O objetivo desta revisão foi descrever os principais resultados de estudos sobre preconceitos, estereotipia e discriminação com relação à idade (ageismo) no contexto da pandemia da covid-19.

## MÉTODOS

A revisão integrativa de literatura consiste na abordagem metodológica empregada para fornecer conhecimentos produzidos sobre uma determinada temática, de maneira sistemática, ordenada e abrangente. Organiza-se em seis fases: identificação do tema, hipótese ou questão de pesquisa; identificação de critérios pré-estabelecidos de busca na literatura; definição das informações a serem extraídas dos estudos selecionados/categorização dos estudos; avaliação crítica dos estudos incluídos; interpretação dos resultados; e apresentação da revisão/síntese do conhecimento^23^.

Neste estudo, realizou-se uma busca na literatura sobre a discriminação etária (ageismo ou etarismo) contra idosos no contexto da pandemia da covid-19, considerando periódicos nacionais e internacionais. As estratégias de busca eletrônica foram conduzidas por dois pesquisadores independentes, entre o período de 01 de maio a 15 de junho de 2020, nas seguintes bases de dados: *Medical Literature Analysis and Retrieval System Online* (MEDLINE/PubMed), *Web of Science* (Thompson Reuters), *Scopus* (Elsevier Science), Lilacs e da *Scientific Eletronic Library Online* (SciELO). Um terceiro pesquisador foi consultado para opinar sobre a inclusão ou não das publicações selecionadas, visando solucionar divergências entre os dois pesquisadores.

Os descritores foram utilizados de acordo com o *Medical Subject Heading* (MeSH) e seus equivalentes na língua portuguesa, estabelecidos pelos Descritores em Ciências da Saúde (DeCS). Os termos foram combinados utilizando os operadores booleanos “*AND*” e “*OR*” para compor as estratégias de busca, elaboradas para cada base de dados. Os seguintes termos foram utilizados como descritores durante as buscas nas bases de dados: covid-19 *OR* 2019 *novel coronavirus disease OR* covid19 *OR* covid-19 *pandemic OR* SARS-CoV-2 *infection OR* covid-19 *virus disease OR* 2019 *novel coronavirus infection OR* 2019-nCoV *infection OR coronavirus disease* 2019 *OR coronavirus disease*-19 *OR* 2019-nCoV *disease OR* covid-19 *virus infection AND Ageism OR Age Discrimination OR Age Discriminations OR Discrimination, Age OR Discriminations, Age*.

Os critérios de inclusão foram as publicações cujo objeto de estudo era o ageismo na população idosa (pessoas com idade ≥ 60 anos) e os reflexos na pandemia da covid-19. No presente estudo não houve restrição de ano, desenho metodológico ou idioma das publicações sobre o tema. Foram excluídas publicações que tratavam do ageismo em grupos etários inferiores a 60 anos de idade ou não se relacionavam à pandemia da covid-19. As publicações que se apresentaram em mais de uma base de dados foram consideradas apenas uma vez.

## RESULTADOS

A busca inicial nas plataformas de pesquisa identificou 43 publicações com base no título. Desses, 21 manuscritos foram eliminados (com base no título e no resumo) por serem estudos duplicados ou não relacionados ao tema de interesse. Assim, foram selecionados 22 estudos para a leitura na íntegra e, dentre esses, um artigo foi excluído depois da consulta do terceiro pesquisador por não abordar a temática ageismo na pandemia como foco principal. Deste modo, 21 artigos foram utilizados nesta revisão integrativa. Dezoito artigos foram encontrados na MEDLINE/Pubmed, sete na Web of Science, quatro na BVS/Lilacs e três na Scopus. Alguns artigos estavam publicados em duas ou mais bases de dados. A Figura 1 exibe o fluxograma relativo às ações do processo de seleção das publicações.

**Figura:** Fluxograma do processo de seleção dos estudos para a revisão integrativa sobre ageismo no contexto da pandemia covid-19.

A síntese das publicações contempladas nesta revisão, de acordo com a base de dados em que estava disponível, o periódico, o(s) autor(es), ano de publicação, título, tipo de estudo, considerações/objetivos e os resultados de interesse, é mostrada no Quadro 1. A maioria dos artigos (90,4%; n=19) foi publicada em inglês, os demais em espanhol (n = 1) e em português (n=1). De modo geral, as publicações identificadas eram de natureza teórica com abordagem crítico-reflexiva, sendo 90,5% artigos opinativos (n = 19) e 9,5% de pesquisa (n = 2).

**Quadro:** Revisão integrativa das publicações sobre o ageismo contra idosos no contexto da pandemia causada pelo SARS-CoV-2 (covid-19).

Base de dados	Periódico	Autor(es), ano	Título	Tipo	Objetivos	Resultados^a^ / Conteúdos^b^
PubMed, *Web of Science*	*Journal of Clinical Nursing*	Brooke et al., 2020^18^	*Older people and COVID-19: isolation, risk and ageism*^b^	Editorial	Trata do isolamento social, riscos e ageismo durante a covid-19	Discursos preconceituosos, negativos e implicitamente desvalorizadores contribuem para que idosos desenvolvam sentimentos de inutilidade, de serem onerosos e de não terem valor, provocando restrição das oportunidades sociais, tornando os idosos vulneráveis a desfechos sociais e de saúde negativos, particularmente isolamento e solidão.
PubMed, BVS, Scopus, *Web of Science*	*Journal of the American Medical Directors Association*	Cesari et al., 2020^24^	*COVID-19 in Italy: Ageism and decision making in a pandemic*^b^	Editorial	Criticam as recomendações éticas e clínicas acerca da alocação de tratamento intensivo, utilizando o critério etário nas situações de recursos limitados com a covid-19 na Itália.	Condutas baseadas apenas no número de anos vividos configura-se como ageismo. São necessários parâmetros mais robustos do que a idade, para orientar a tomada de decisões críticas e rápidas na atenção à saúde dos idosos, entre eles a fragilidade, as comorbidades e o status funcional.
PubMed, *Web of Science*	*Journal of the American Geriatrics Society*	Jimenez-Sotomayor et al., 2020^25^	*Coronavirus, ageism, and twitter: an evaluation of tweets about older adults and COVID -19*^a^	Estudo qualitativo	Analisaram os *tweets* relacionados aos idosos e covid-19 para identificar o conteúdo de discriminação etária.	Quase um quarto dos tweets analisados (21,1%; n=74) tinha conteúdos de discriminação etária ou eram potencialmente ofensivos aos idosos, designados como “boomers”. Divulgava-se que a vida de idosos era menos valiosa, ou então o perigo da covid-19 era minimizado devido ao fato de prejudicar principalmente os idosos.
PubMed	*Geriatrics*	Petretto et al., 2020^26^	*Ageing and COVID-19: what is the role for elderly people?*^b^	Editorial	Aborda o papel dos idosos no contexto da covid-19 e destacam os riscos do ageismo a partir da experiência na Itália.	A maior vulnerabilidade dos idosos à infecção pelo coronavírus aumenta o risco de ageismo. O desempenho ativo de papéis durante a pandemia reduz esse risco. Os idosos podem enfrentar grandes barreiras no acesso ao serviço e apoio à saúde, baseados no ageismo. É importante proteger os idosos da infecção, mas também é muito importante respeitá-los e apoiá-los nessa situação complexa.
PubMed, *Web of Science*	*Journal of Aging & Social Policy*	Morrow-Howell et al., 2020^27^	*Recovering from the COVID-19 Pandemic: a focus on older adults*^b^	Artigo – Perspectiva	Discute, com foco nos idosos, os desafios (incluindo o ageismo) e as oportunidades decorrentes da pandemia da covid-19.	A pandemia da covid-19 evidenciou que o ageismo e os estereótipos de velhice contribuíram para o abandono das pessoas idosas e para a negligência em relação às suas necessidades. O uso de memes e hashtags que estimulam a ideia de eliminar os idosos ou tirá-los do caminho servem para depreciar os idosos e para valorizar o vírus, por ajudar a reduzir o gasto público com esse segmento etário caracterizado como improdutivo, dependente e dispendioso.
PubMed	*Age and Ageing*	Fraser et al., 2020^28^	*Ageism and COVID-19: what does our society’s response say about us?*^b^	Comentário	Destacam o ageismo que surgiu durante a pandemia da covid-19 e discute como as pessoas idosas são deturpadas e desvalorizadas no discurso público atual em torno da pandemia.	O discurso público atual em torno da covid-19 deturpa e desvaloriza os idosos. A pandemia inicialmente não foi levada a sério e o discurso público a apresentou como perigosa apenas para os idosos. Possivelmente essa narrativa explica resistência em seguir as diretrizes de saúde pública. O ageismo atingiu um novo nível com as memes e hashtags que falam em matar ou eliminar os idosos, que são representados como seres vulneráveis e desamparados, cuja morte pelo vírus já era prevista e considerada inevitável.
PubMed, Scopus	*Asian Journal of Psychiatry*	Banerjee, 2020^29^	*‘Age and ageism in COVID-19’: elderly mental health-care vulnerabilities and needs*^b^	Nota para editor	Abordam o ageismo e a vulnerabilidade da idade na pandemia da covid-19 e a necessidade de cuidados da saúde mental e bem-estar dos idosos.	O impacto da pandemia pode ser maior nos idosos pois eles têm ciência da sua vulnerabilidade, o que pode levar à autonegligência. O ageismo pode levar à marginalização e ao abuso do idoso, gerando dependência funcional e diminuição do bem-estar.
PubMed	*Journal of Gerontological Social Work*	Berg-Weger et al., 2020^30^	*COVID-19 Pandemic: Workforce implications for gerontological social work*^b^	Artigo	Abordam quatro questões que se destacaram com a pandemia - o ageismo, a tecnologia, o isolamento social e solidão e a prática interprofissional, considerando-se as implicações da força de trabalho para o trabalho social gerontológico com a covid-19.	O ageismo tem sido demonstrado ao longo da pandemia, incluindo a falta de protocolos para os idosos, a falta de conteúdos de geriatria nos currículos das profissões que prestam assistência aos idosos, as desigualdades na alocação de recursos necessários, as referências depreciativas feitas à pandemia covid-19 nos idosos e o alívio devido à mortalidade acometer mais essa faixa etária.
PubMed, *Web of Science*	*Journal of Gerontological Social Work*	Swinford et al., 2020^31^	*Applying gerontological social work perspectives to the coronavirus pandemic*^b^	Comentário	Analisaram três perspectivas gerontológicas da assistência social na pandemia da covid-19.	A heteregoneidade da população idosa desafia o ageismo e os estereótipos etários que surgiram com a covid-19. As ideias do ageismo são baseadas em estereótipos etários prejudiciais que reduzem um grupo extremamente heterogêneo a uma única coorte. É possível rejeitar o discurso ageista e reforçar os argumentos para apoiar os idosos e os trabalhadores, voluntários e cuidadores, assim como promover ativamente os esforços para expandir a solidariedade entre as gerações.
PubMed	*International Psychogeriatrics*	Ayalon, 2020^17^	*There is nothing new under the sun: ageism and intergenerational tension in the age of the COVID-19 outbreak*^b^	Comentário	Aborda a questão da relação entre o ageismo e a tensão intergeracional na pandemia da covid-19.	Durante a pandemia os idosos se destacaram como os principais afetados e menos obedientes às práticas sociais atuais. Todos os idosos foram colocados em um mesmo grupo homogêneo e vulnerável, ignorando a hetereogeneidade do envelhecimento. Isso reforça o ageismo e faz com que cresçam as desavenças entre as diferentes gerações.
PubMed	*The Journals of Gerontology*	Ayalon et al., 2020^32^	*Aging in times of the COVID-19 pandemic: avoiding ageism and fostering intergenerational solidarity*^b^	Editorial	Comenta como o discurso ageista pode afetar todas as gerações	A pandemia suscitou um surto paralelo de ageismo. A mídia destacou os idosos como um grupo de pessoas frágeis, impotentes e incapazes de contribuir para a sociedade. A incorporação desses estereótipos pode ser prejudicial aos idosos e aos jovens durante seu próprio envelhecimento. É importante que o distanciamento físico não seja também social e que sejam fortalecidas as relações entre as gerações. Não se deve pensar em grupo de risco apenas pela idade, sem levar em consideração outros fatores, como doenças crônicas e fragilidade.
PubMed, BVS, Scopus	*British Medical Journal*	Archard et al., 2020^33^	*Is it wrong to prioritise younger patients with covid-19?*^b^	Nota para o editor (Opinião)	Explica razões pelas quais a idade não pode ser um fator determinante para decidir qual vida deve ser priorizada.	Não seria correto decidir quem deve viver apenas pela idade pois todos devem ter a oportunidade de levar a vida por uma determinada duração. Além disso, a vida de um idoso não vale menos que a vida de um jovem. O ponto chave é utilizar a ética para não tomar atitudes discriminatórias e ageistas segundo as quais idosos têm menos valor ou importância do que jovens.
PubMed	*Journal of Aging & Social Policy*	Ehni et al., 2020^34^	*Six propositions against ageism in the COVID-19 pandemic.* ^b^	Artigo (categoria outros)	Oferece seis propostas baseadas no conhecimento gerontológico e na ética do envelhecimento contra o ageismo, presentes nas reações atuais à pandemia do covid-19.	O foco em comentários sobre os idosos desenvolverem a forma mais grave da covid-19 e possuírem maior risco de óbito tornou-se evidente na pandemia. Muitas atitudes na pandemia são baseadas em estereótipos negativos de saúde e funcionamento dos idosos. Isso desvaloriza a vida da pessoa mais velha e proporciona aumento do ageismo, portanto, os autores destacam seis propostas que podem mudar esse comportamento. As propostas serão apresentadas mais adiante.
PubMed, BVS	*Revista Española de Geriatria y Gerontologia*	Tarazona-Santabalbina et al., 2020^35^	*COVID-19, adulto mayor y edadismo: errores que nunca han de volver a ocurrir [COVID-19, older adults and ageism: mistakes that should never happen again]*^b^	Editorial	Discorre sobre atitudes ageistas que ocorrem durante a pandemia e o que deve ser feito para saná-las.	As decisões tomadas durante essa emergência de saúde não justificam a desvalorização da pessoa idosa. Os profissionais da geriatria e gerontologia deveriam se unir para gerar mais informações a todos e acabar com o ageismo. Além disso, os idosos devem ter acesso aos testes e equipe de saúde especializada para um atendimento adequado, dessa forma tais erros cometidos não acontecerão nos próximos meses.
PubMed	*British Journal of Anaesthesia*	Savulescu et al., 2020^36^	*Equality or utility? Ethics and law of rationing ventilators*^b^	Editorial	Explica sobre os termos éticos igualitarismo e utilitarismo, como eles se aplicam no cenário da pandemia para a tomada de decisão na alocação de cuidados intensivos e ventilação mecânica e decisões sobre qual vida manter.	Em respeito à ventilação mecânica, as decisões devem ser tomadas pela equipe de saúde junto ao paciente. Não podem ser baseadas apenas na idade ou incapacidade, pois isso pode ser preconceituoso. Os autores sugerem utilizar o “utilitarismo preventivo”, forma que daria algum peso para garantir a igualdade, reconhecendo que as pessoas não devem ser discriminadas arbitrariamente. Dessa forma o maior número de pessoas seria beneficiado com o maior bem, porém tratadas de forma igualitária.
PubMed	*Journal of Aging & Social Policy*	Previtali et al., 2020^37^	*Not only virus spread: The diffusion of ageism during the outbreak of COVID-19.* ^b^	Artigo	Aborda a difusão do ageismo durante a pandemia da covid-19.	O ageismo leva a consequências prejudiciais e a pandemia aumentou a sua incidência. Apesar da covid-19 afetar pessoas de todas as idades, os idosos têm sido destaque nas mídias. Alguns comentários em mídias sociais reforçam o ageismo, porém, ao contrário do que tem sido divulgado, os idosos são muito ativos na sociedade, são afetados pelo isolamento social. As práticas do ageismo durante a pandemia reforçam estereótipos e violam os direitos humanos, devendo haver esforço coletivo para que isso acabe.
PubMed	*European Cardiology Review*	Martínez-Sellés et al., 2020^38^	*Ethical issues in decision-making regarding the elderly affected by coronavirus disease 2019: an expert opinion*^b^	Artigo de opinião	Opina sobre a tomada de decisões na covid-19 em relação à população idosa.	Visto que os idosos sofrem maior risco de contaminação pelo coronavírus, essa população deveria ser priorizada nas medidas de prevenção. Não se pode tomar uma decisão utilizando apenas a idade e sim naqueles com maior probabilidade de sobreviver. Os idosos sofrem riscos físicos e psicológicos com o isolamento social, não só em casa, mas também nos hospitais, instituições e até nos velórios.
PubMed	*Journal of Aging & Social Policy*	Reynolds, 2020^39^	*The COVID-19 pandemic exposes limited understanding of ageism*^b^	Artigo	Aborda a falta de conhecimento sobre ageismo e seus impactos no envelhecimento.	A falta de conhecimento sobre o que é ageismo ficou muito evidente com a covid-19. O caso do tenente-governador do Texas falando que abriria mão da própria vida para salvar a geração de seus netos ilustra dimensões e construções do ageismo no contexto do covid-19. Muitos comentários não intencionais surgem inclusive de profissionais de saúde e cuidadores. Devemos tentar implementar esse conceito biopsicossocial em diferentes espaços.
BVS	Cogitare Enfermagem	Hammerschmidt, et al., 2020^40^	Saúde do idoso em tempos de pandemia COVID-19 ^b^	Comunicação livre	Abordam de forma reflexiva e crítica aspectos relacionados à saúde do idoso nos tempos de pandemia da covid-19.	A pandemia provocou o afloramento do ageismo. As ações de proteção ao idoso durante a pandemia reforçaram estereótipos do envelhecimento. Tal situação pode afetar negativamente relações familiares e promover conflitos intergeracionais. A pandemia destacou a importância da atenção à saúde do idoso, mas também reforçou comportamentos agesitas, ridicularização e julgamentos. As ações de distanciamento físico devem manter a independência e a autonomia do idoso.
*Web of Science*	*Journal of Loss and Trauma*	Rahman, et al., 2020^41^	*Defining a ‘risk group’ and ageism in the era of COVID-19* ^b^	Artigo	Trata da incidência cumulativa da covid-19. Aborda a classificação de grupo de maior risco, o que não é apenas questionável, mas também problemático.	Rotular os idosos como grupo de risco apenas pela idade é uma forma de ageismo que pode acelerar o isolamento social e aumentar os níveis de sofrimento psicossocial deve ser levada em conta não só a idade, mas também fatores sociais, culturais e contextuais. As plataformas de mídia social devem ser usadas de forma positiva, divulgando notícias verdadeiras e evitando o pânico. A heterogeneidade do envelhecimento deve ser reconhecida e as atitudes positivas a serem tomadas em relação aos idosos deve começar dentro da nossa própria casa e ir até as mídias eletrônicas.
*Web of Science*	International Journal of Mental Health and Addiction	Flett, et al., 2020^42^	*Aging and feeling valued versus expendable during the COVID-19 pandemic and beyond: a review and commentary of why mattering is fundamental to the health and well-being of older adults*^a^	Revisão e comentário	Analisaram o papel protetor dos sentimentos de importância entre os idosos em tempos típicos e atípicos, estes associados à pandemia da covid-19.	Apresentam o conceito de *“mattering”* e suas particularidades na população idosa. Todas as pessoas sentem o desejo de sentirem importantes e valorizadas. Na pandemia, tal fato se ressalta ainda mais nos idosos. Com as recomendações e comentários feitos, muitos idosos podem se sentir ainda mais vulneráveis e com falta de cuidado. Além disso, há o isolamento social que prejudica ainda mais a saúde da população idosa. Deve-se ter cuidado com os comentários estereotipados sobre envelhecimento e medidas devem ser criadas para preservar a saúde física e mental dos idosos.

^a^ Artigos de pesquisa.

^b^ Artigos opinativos.

Os artigos de opinião destacaram a importância dos pesquisadores da área se posicionarem acerca de uma questão bioética, cultural, social e ética envolvendo o mundo todo. Os autores se posicionam não só como profissionais de saúde, mas também como seres humanos diante de seus iguais em um problema que marcará o destino de muitas pessoas. O novo coronavírus nos traz um novo paradigma para a busca de conhecimento, bem comum e solidariedade.

Observou-se que a maioria das publicações^18,24,27^destacam que o ageismo ficou mais evidente durante a pandemia da covid-19, gerando diversos impactos negativos para os idosos. Brooke e Jackson^18^ enfatizam que o isolamento prolongado pode contribuir para a solidão, diminuição da mobilidade, aumento da fragilidade e depressão em idosos, além disso, os discursos “ageistas” podem aumentar o abandono dos mais longevos^18^. Banerjee^29^ sugere que, em idosos, a negligência, a solidão, a depressão, a ansiedade, o isolamento e os abusos são males associados ao distanciamento social durante a pandemia, podendo ser mais problemáticos naqueles institucionalizados, em que as medidas de distanciamento e higiene podem não ser adequadas. Morrow-Howell et al.^27^ ressaltam que os idosos podem ter efeitos emocionais duradouros devido ao aumento do isolamento e da ansiedade.

Jimenez-Sotomayor et al.^25^ analisaram *tweets* e identificaram que 21,1% deles continham comentários com algum preconceito etário ou menosprezavam a gravidade da covid-19 por considerarem que atingia apenas os idosos. Outros estudos^17,18,28^ também identificaram o ageismo presente nas redes sociais com o uso da *hashtag #boomerremover*, normalmente acompanhada de imagens depreciativas e piadas relacionadas aos idosos. Algumas publicações^24,33,36,40^ abordaram de forma crítica a destinação de recursos, de cuidados intensivos, de ventilação mecânica e/ou tomada de decisões baseadas exclusivamente no critério etário.

O ageismo e as relações intergeracionais também foram abordadas^17,32^. Com os discursos “ageistas” cada vez mais presentes na mídia durante a pandemia, houve a acentuação do conflito entre pessoas de gerações diferentes. Dividir a sociedade por faixas etárias torna ainda mais difícil o enfrentamento dos idosos diante de um fenômeno natural devastador como a pandemia^17^. A ênfase na idade como fator determinante da gravidade da covid-19 foi propagada também por profissionais da saúde, evidenciado a falta de conhecimento dos mesmos e da população geral sobre o ageismo. Nesse caso o ageismo raramente é feito de forma intencional, porém, pode afetar negativamente a vida dos idosos. Dessa forma, é importante que se entenda o ageismo como um conceito biopsicossocial e sua definição seja disseminada em diferentes espaços^41^

## DISCUSSÃO

Os principais achados desta revisão mostram que o ageismo se tornou presente em vários aspectos na vida dos idosos durante a pandemia. Embora todas as pessoas possam ser afetadas pela doença (covid-19), os idosos estão no centro da mídia e na maioria das discussões acerca da temática.

Um estudo^42^ identificou que as experiências de ageismo foram mais comuns em idosos e adultos jovens, comparativamente às pessoas de meia-idade. Enquanto os adultos jovens referiram sofrer ageismo mais frequentemente no ambiente de trabalho, em idosos e indivíduos de meia-idade foi mais comum quando procuravam mercadorias e serviços. Curiosamente, os idosos não citaram familiares como principais praticantes de ageismo, valendo ressaltar que, geralmente, quando há algum comentário desse tipo por um familiar, ele é interpretado como menos agressivo. Considerando-se o tipo de experiência, os jovens relataram a falta de respeito, já os adultos de meia-idade e os idosos citaram suposições sobre capacidade social e física^42^.

O ageismo pode ocorrer em nível estrutural, em que as instituições sociais reforçam o preconceito sistemático contra os idosos e em nível individual, no qual os indivíduos têm opiniões negativas sobre o processo de envelhecimento. Revisão sistemática, que incluiu 422 estudos (mais de 7 milhões de participantes), identificou que em 95,5% dos estudos analisados o ageismo levou a piores resultados na saúde de idosos. Observou-se ainda uma prevalência maior do efeito negativo do ageismo na saúde em países menos desenvolvidos quando comparados aos mais desenvolvidos (p = < 0,001). O ageismo foi associado com piora na saúde em todos os domínios analisados^43^.

Ressalta-se que, durante a pandemia, o ageismo pode ser mais frequente, tendo em vista que muitos idosos permanecem dentro de suas casas dependendo da ajuda e prestação de serviços de outras pessoas para, por exemplo, obter itens básicos. Dessa forma, pode ser dado um foco errôneo nas características físicas dos idosos como um grupo heterogêneo de pessoas frágeis e dependentes^36^.

Estudo realizado com idosos na Austrália^44^, identificou que aqueles com algum tipo de incapacidade eram mais propensos a relatar discriminação do que idosos sem incapacidade ou com condições crônicas de saúde. Os indivíduos que sofreram preconceito apresentaram menores escores de autoeficácia e satisfação com a vida, denotando efeitos negativos da discriminação na vida das pessoas idosas^44^.

Previtali et al.^37^ enfatizam que, embora seja observada relação entre a presença de doenças crônicas e a idade, ser cronologicamente idoso não significa ser vulnerável, em estado precário de saúde ou menos valioso. Além disso, a ideia de que a idade cronológica define objetivamente os grupos, negligenciando suas diferenças internas, é uma suposição que configura ageismo, pois apoia preconceitos, estereótipos e discriminação com base na idade^37^.

Alguns estudos indicam o ageismo nos serviços de saúde durante a pandemia da covid-19, principalmente na distribuição de recursos, como a destinação de ventiladores mecânicos prioritária para jovens, em relação aos idosos. Na Itália, devido ao elevado e crescente número de pacientes com covid-19, o critério de idade começou a ser implicitamente adotado no algoritmo decisório para a alocação dos recursos escassos^24^. Segundo Ouchida^45^, a forma como os profissionais de saúde consideram o envelhecimento e o idoso pode determinar o atendimento e tratamento do mesmo. Ademais, a destinação de recursos baseada apenas na idade caracteriza-se como ageismo, pois, em situações críticas, devem ser considerados outros parâmetros, como condições clínicas, fragilidade, estado funcional e comorbidades^24^. Todos têm direito à vida e as decisões rápidas deveriam ser tomadas pela equipe em conjunto com o paciente e família^33,36^.

O ageismo pode ser implícito ou explícito e muitas vezes pode não ser reconhecido. Considerando-se o aumento da população idosa, torna-se cada vez mais importante a comunicação adequada entre profissionais de saúde e indivíduos, a melhor compreensão da heterogeneidade do envelhecimento e a renúncia de estereótipos relacionados à velhice^46^. Cesari et al.^24^ sugerem que médicos familiarizados com os princípios de geriatria e gerontologia apoiem o desenvolvimento de recomendações mais contemporâneas, identificando maneiras válidas e eficientes de avaliar morbidades e status funcional nos diferentes contextos e especialidades.

As publicações apontam que o isolamento social impactou negativamente na vida dos idosos. Plagg^47^ avaliou os benefícios e danos decorrentes do isolamento social prolongado para os idosos e destacam que, apesar do objetivo principal ser evitar ou diminuir a propagação do vírus, a situação eleva o risco de doenças neurológicas, cardiovasculares, depressão, declínio cognitivo e mortalidade^45^. Portanto, caso prolongado o isolamento social, medidas para reduzir os possíveis danos devem ser tomadas. Além disso, o distanciamento não deve ser entendido como o fim das relações sociais e das redes de apoio, e os profissionais de saúde, a sociedade e a família devem trabalhar juntos para que o idoso continue com a sensação de pertencimento.

Com as recomendações do distanciamento físico e isolamento domiciliar, as mídias sociais surgiram como principal alternativa para que o indivíduo possa manter algum contato humano, ainda que indireto. Além disso, a cobertura da mídia sobre a pandemia da covid-19 desempenhou papel fundamental na disseminação rápida de pesquisas científicas e informações por autoridades sanitárias. Em contrapartida, a propagação de *fake news, memes* depreciativos, opiniões ofensivas (como o uso da hashtag *#boomerremover*) evidenciaram o ageismo vigente na sociedade, que erroneamente prega o coronavírus como uma doença “de velho” e potencializa o teor discriminatório contra aqueles de mais idade^25,27^. A criação de conteúdo confiável, de alta qualidade e que enfrente o ageismo faz-se necessária a fim de reduzir os efeitos maléficos dos estereótipos negativos do envelhecimento na saúde e no bem-estar da população idosa^25,48^.

A tensão intergeracional – caracterizada como o conflito entre pessoas de diferentes gerações – manifestou-se em forma de raiva e ódio nas redes sociais por causa da resistência de alguns idosos para usar máscaras ou aderir às medidas de isolamento social. Além disso, foi estabelecida a ideia de que idosos “já viveram suas vidas” e agora é hora de renunciarem, ignorando sua autonomia, independência e desconsiderando suas necessidades sociais^17^. Tais fatos indicam a acentuada diferença e animosidade entre gerações, baseando-se apenas na idade como um marcador de risco e de letalidade da covid-19^17^.

Estereotipar o idoso como indivíduo frágil e dependente pode causar problemas em todas as gerações, pois os mais jovens interiorizam essa imagem e a projetarão para si em seu envelhecimento. Não é apenas a idade que tornam os indivíduos mais vulneráveis ao covid-19, existem outros fatores como a presença de doenças crônicas e comorbidades^32^. A solidariedade entre gerações é importante para maximizar o apoio, a conexão e a rede de suporte social dos idosos durante a pandemia^28^. Revisão sistemática e metanálise, que incluiu 63 estudos (6.124 participantes), identificou que as intervenções focadas em educação, contato intergeracional e educação, combinada ao contato intergeracional, associaram-se à redução do ageismo^49^.

Outro agravante é o comportamento social na velhice, muitas vezes, caracterizado pela redução das redes sociais e diminuição da participação em atividades sociais^50^. Ainda, o acesso ou a capacidade limitada de uso das tecnologias digitais pelos idosos pode impedi-los ou dificultar a obtenção de bens, serviços e apoio social necessários durante a pandemia, deixando-os mais vulneráveis ao isolamento, à depressão e à solidão^32^. Previtali et al.^37^ apontam que o acesso à tecnologia, bem como à alfabetização digital, demonstrou ser um elemento-chave na capacidade de lidar com os desafios impostos pela quarentena.

É muito importante proteger os idosos da covid-19, mas também é importante respeitá-los e apoiá-los nessa situação complexa^26^. Com o surgimento de numerosos dilemas ageistas e conflitos morais referentes ao valor da vida das pessoas idosas em meio a pandemia, destacam-se os seis pontos elaborados por Ehni et al.^34^, baseados em conhecimentos gerontológicos e na ética do envelhecimento para o combate do ageismo presente nas reações à pandemia: 1) os idosos são altamente heterogêneos - sua saúde e funcionamento são melhores do que os estereótipos negativos sugerem; 2) limites de idade para terapia intensiva e outras formas de assistência médica são inapropriados e antiéticos; 3) a visão deficitária da velhice é perigosa para os idosos e sociedades em geral - a solidariedade entre gerações deve ser fortalecida; 4) resistir à suposição de uma atitude paternalista em relação aos idosos; 5) a crise da covid-19 exige o uso de modernas tecnologias da informação e comunicação entre os idosos e; 6) a crise do covid-19 não apenas exige o melhor da virologia, mas também o melhor da gerontologia para orientação política e a compreensão das consequências em geral.

Dentre as limitações deste estudo, deve-se considerar que, globalmente, o ageismo é uma temática ainda pouco discutida no contexto da pandemia da covid-19, o que pode explicar, mesmo que parcialmente, o número reduzido de publicações encontradas sobre o tema e a predominância de artigos opinativos (editoriais e comentários). Contudo, ressalta-se a natureza critico-reflexiva sustentada em importantes pressupostos teóricos que permitem analisar diversos pontos críticos socioculturais e de abrangência em saúde pública.

De modo geral, os resultados desta revisão integrativa demonstram um retrato do contexto e fenômeno de interesse dos estudos primários, trazendo à tona esse tema tão importante e ainda pouco discutido em relação à pandemia no Brasil e no mundo.

Os discursos “ageistas” podem causar impactos sociais e psicológicos negativos na vida dos idosos. Faz-se necessário aumentar os esforços para a redução do ageismo, bem como a difusão de informações sobre essa prática tão prejudicial. Observa-se a importância do desenvolvimento de políticas públicas e estudos científicos envolvendo o tema a fim de promover a construção de uma sociedade mais justa e igualitária, com solidariedade intergeracional e respeito aos direitos e a vida das pessoas idosas.
